# Paratesticular Fibrous Pseudotumor in a Pediatric Patient: A Case Report

**DOI:** 10.7759/cureus.71453

**Published:** 2024-10-14

**Authors:** Inês Coelho Mogárrio, Filipa Jalles, Maria Knoblich, Rui Alves

**Affiliations:** 1 Pediatric Surgery, Hospital Dona Estefânia, Unidade Local de São José, Lisboa, PRT; 2 Pediatric Surgery, Hospital Central do Funchal, Funchal, PRT

**Keywords:** paratesticular fibrous pseudotumors, pediatric, surgery, testicular mass, urology

## Abstract

Paratesticular fibrous pseudotumors are rare benign tumors. This case reports paratesticular fibrous pseudotumors in a very young patient. A previously healthy 16-month-old boy was seen due to a growing scrotal mass. On clinical examination, there was a painless, multinodular scrotal mass. Tumor markers were normal, and a testicular ultrasound with Doppler revealed a solid, avascular, and hypoechoic mass (50x20 mm). The patient underwent excision of the scrotal mass and adjacent skin. The histological analysis revealed a paratesticular fibrous pseudotumor. Definitive treatment is surgical excision, and if there is any concern for malignancy, an extemporaneous examination should be done to confirm the diagnosis. The prognosis with fibrous pseudotumors is excellent.

## Introduction

Paratesticular fibrous pseudotumors are rare benign tumors in pediatric age, especially in prepubertal patients [1]. They represent about 6% of paratesticular masses [2] and have a peak incidence in the third decade of life [1,2]. About 50% of cases are associated with hydrocele [1]. An entity first described by Astley Cooper in 1830, it is thought to be a reactive process to previous trauma, inflammation or infection [2]. Although they are benign lesions, they can mimic malignant lesions, such as leiomyosarcoma, rhabdomyosarcoma, and desmoplastic small round-cell tumors [3]. For this reason, orchiectomy was performed in the past [2-4].

## Case presentation

**Figure 1:**
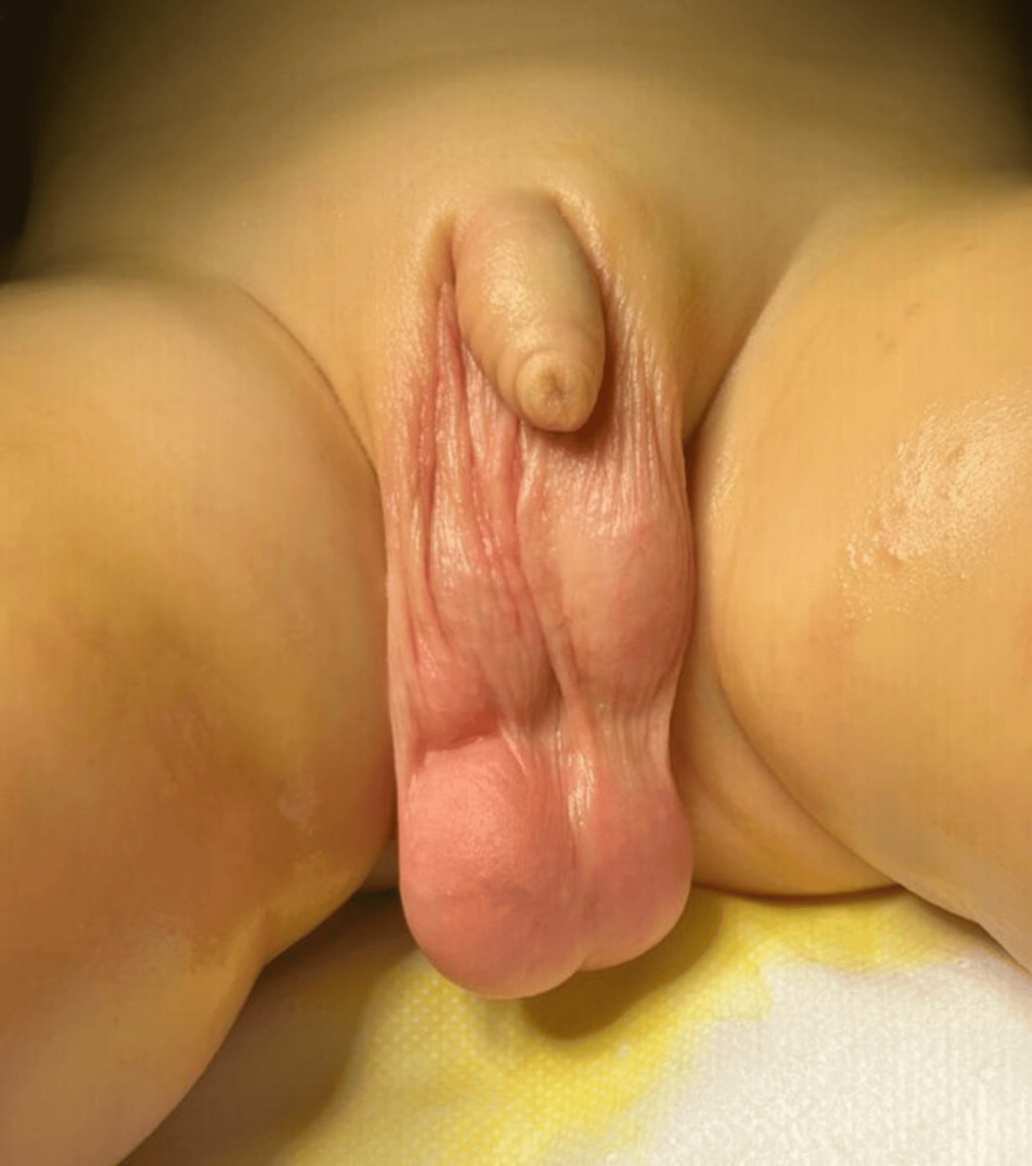
Patient image at the time of presentation Multinodular, well-circumscribed scrotal mass, caudally to both testicles and apparently more adjacent to the right testicle.

A previously healthy 16-month-old boy was seen in our outpatient clinic due to a growing scrotal mass that had been noted six months before (Figure 1).

**Figure 2:**
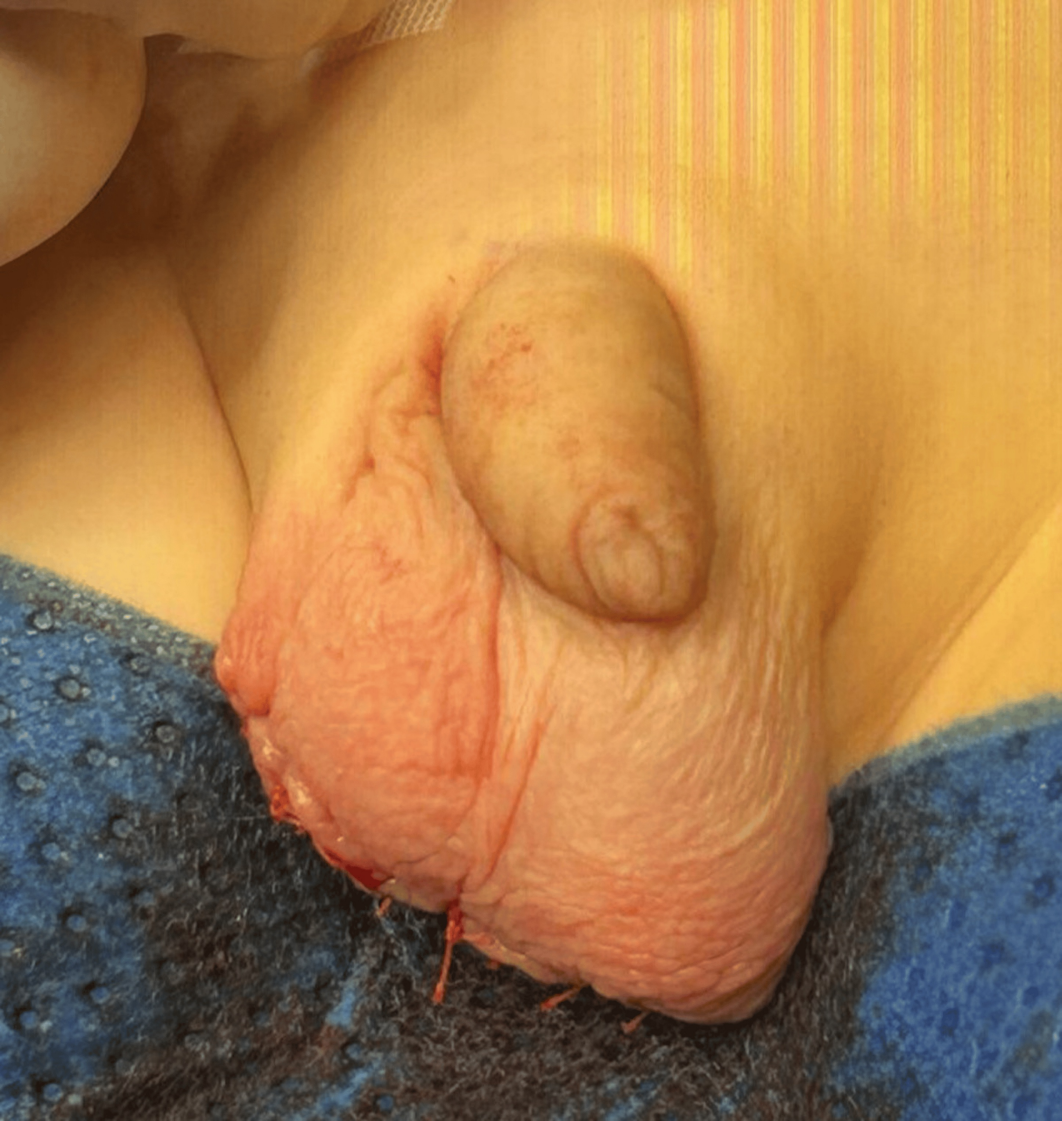
Excision of the scrotal mass and adjacent skin through scrotal exploration

**Figure 3:**
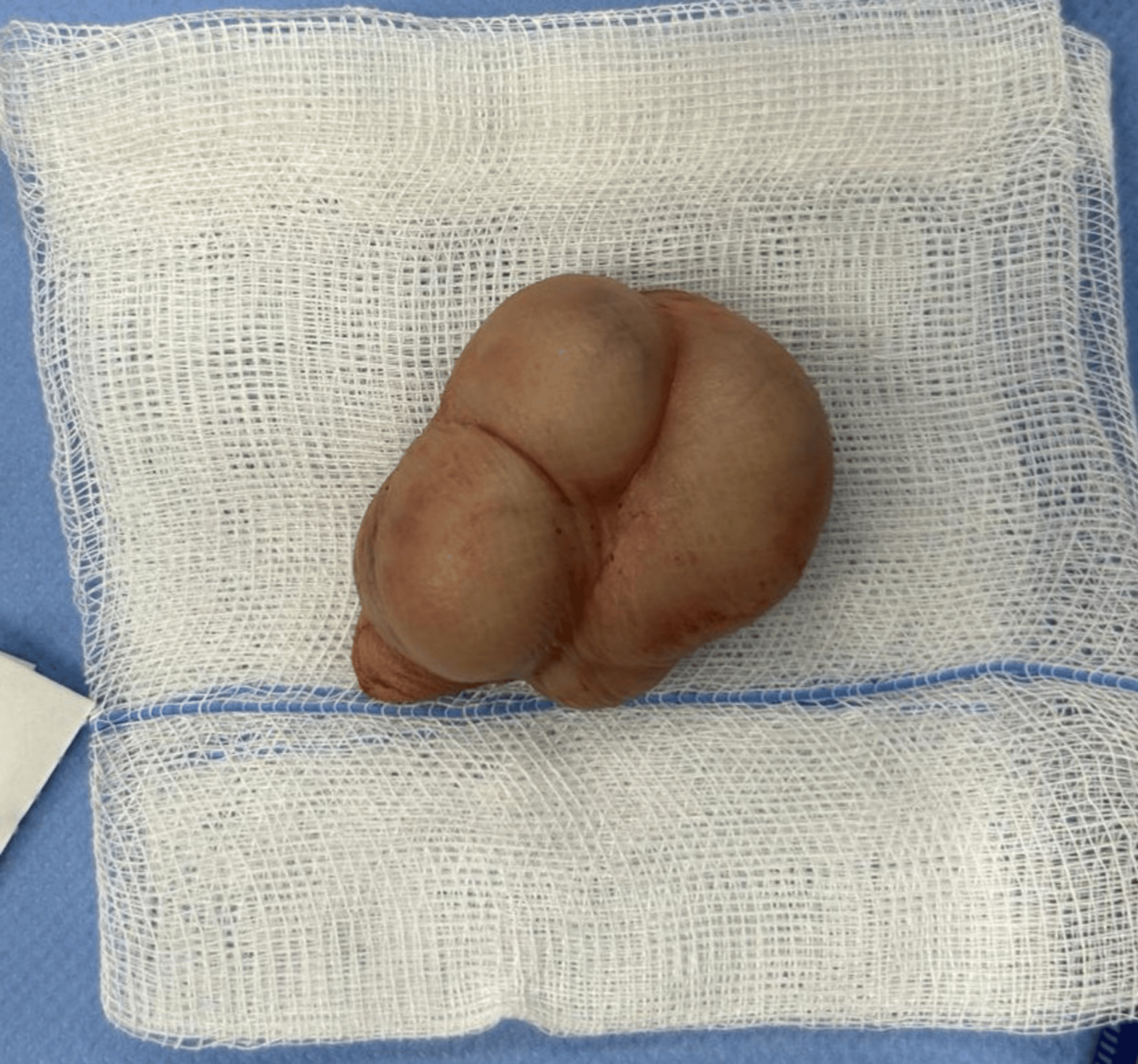
Scrotal mass and adjacent skin

He denied any pain in the scrotum, and there was no history of trauma. On clinical examination, his testes were descended bilaterally, of symmetrical size and consistency, and no hydrocele or hernia was appreciated. There was a painless, multinodular, well-circumscribed, mobile lesion caudally to both testicles, and it appeared related to the right testicle. No palpable inguinal masses were found. His α-fetoprotein and β-human chorionic gonadotropin levels were within the normal range, and a testicular ultrasound with Doppler revealed a solid, avascular, and predominantly hypoechoic mass (50x20 mm) with lobulated contours and without calcifications or intra-abdominal extension; testicles were described as normal. The patient underwent excision of the scrotal mass and adjacent skin through scrotal exploration after an unsuccessful right inguinal approach since the mass could not be delivered this way as it was not communicating with the right testis or spermatic cord (Figures 2, 3).

The patient tolerated the procedure well and was discharged home the next day. The histological analysis revealed morphological aspects compatible with a paratesticular fibrous pseudotumor. After 11 months of follow-up, the patient remains with no complaints or signs of recurrence.

## Discussion

Paratesticular fibrous pseudotumors are a rare benign tumor of testicular tunics that most often originates from the tunica vaginalis, occasionally from the epididymis, and rarely from the spermatic cord and tunica albugínea [2-5]. The diagnosis is usually incidental since the only symptom is the slow and progressive growth of the lesion. The differential diagnosis of paratesticular masses is difficult and includes hemangiomas, lipomas, leiomyomas, adenomatoid tumors, and fibrous pseudotumors [1]. It is the second most common paratesticular neoplasm after adenomatoid tumors [2,3]. Its incidence is extremely rare, with 200 cases reported in the literature [5,6], eight of which are of pediatric age. This case is the youngest patient reported in the literature. In the pediatric population, the mean age at diagnosis is 13 years old, with a predominance of the tumor on the right side. The testosterone spike is therefore thought to have some effect on tumor growth [2]. Diagnosis based on clinical status alone is difficult to distinguish from malignant tumors. The investigation should be complemented with tumor markers (α-feto-protein and β-human chorionic gonadotropin), and the recommended imaging test is Doppler ultrasonography [2,7]. When ultrasonography is inconclusive, magnetic resonance imaging may be useful for further tissue characterization [3,6]. Immunoglobulin (Ig)G4-related disease is a heterogeneous entity that includes retroperitoneal fibrosis, sclerosing pancreatitis, sclerosing cholangitis, and Riedel’s thyroiditis [5]. Recent studies have shown a correlation between paratesticular fibrous pseudotumors and IgG4-related sclerosis due to the presence of IgG4-stained plasma cells in the lesions [3,4]. However, only a few cases have been reported in the literature supporting this correlation [5]. In this case, we did not consider the possibility of paratesticular fibrous pseudotumor before surgery, so we did not perform IgG-related laboratory tests. The patient was referred to a rheumatology appointment. Definitive treatment is surgical excision and if there is any concern for malignancy, an extemporaneous examination should be performed to confirm the diagnosis [8]. This should be especially considered when the testicle is involved [1]. Despite the greater ease of a scrotal approach, due to the intrinsic anatomical risk of metastatic seeding in case of malignancies, an inguinal approach is desirable [5]. Prognosis with fibrous pseudotumors is excellent, with no recurrence cases reported [1,4,9], despite the fact that there is no consensus on a follow-up period in the literature [10,11].

## Conclusions

Paratesticular fibrous tumors are rare, especially in pediatric age. Differential diagnosis with malignant neoplasm is difficult, so surgical exploration is imperative to exclude a malignant process. The gold standard treatment is excision and extemporaneous examination, with testicular preservation.
